# Bioactive Compounds from Lemon (*Citrus limon*) Extract Overcome TNF-α-Induced Insulin Resistance in Cultured Adipocytes

**DOI:** 10.3390/molecules26154411

**Published:** 2021-07-21

**Authors:** Valeria Sorrenti, Valeria Consoli, Salvo Grosso, Marco Raffaele, Margherita Amenta, Gabriele Ballistreri, Simona Fabroni, Paolo Rapisarda, Luca Vanella

**Affiliations:** 1 Department of Drug and Health Sciences, University of Catania, 95123 Catania, Italy; valeria_consoli@yahoo.it (V.C.); salvogrosso@outlook.it (S.G.); marco.raffaele@hotmail.com (M.R.); lvanella@unict.it (L.V.); 2 Council for Agricultural Research and Economics (CREA), Research Centre for Olive, Fruit and Citrus Crops, 95024 Acireale, Italy; margherita.amenta@crea.gov.it (M.A.); gabriele.ballistreri@crea.gov.it (G.B.); simona.fabroni@crea.gov.it (S.F.); paolo.rapisarda@crea.gov.it (P.R.)

**Keywords:** lemon extract, adipocytes, insulin resistance, polyphenols

## Abstract

The consumption of plant-based food is important for health promotion, especially regarding the prevention and management of chronic diseases such as diabetes. We investigated the effects of a lemon extract (LE), containing ≥20.0% total flavanones and ≥1.0% total hydroxycinnamic acids, on insulin signaling in murine 3T3-L1 adipocytes treated with TNF-α, which was used to mimic in vitro the insulin resistance condition that characterizes diabetes mellitus. Our results showed LE increased PPARγ, GLUT4 and DGAT-1 levels, demonstrating the potential of this lemon extract in the management of insulin resistance conditions associated with TNF-α pathway activation. LE treatment further decreased the release of interleukin 6 (IL-6) and restored triglyceride synthesis, which is the main feature of a healthy adipocyte.

## 1. Introduction

The worldwide ever-increasing incidence of obesity has brought scientists to focus on understanding adipocyte functions and the biochemical mechanisms by which adipose tissue plays its role as an active endocrine tissue. Adipokines are adipose-derived bioactive peptides involved in the regulation of inflammatory responses and metabolism, showing both pro-inflammatory and anti-inflammatory activities. Among them, tumor necrosis factor α (TNF-α) is the most common pro-inflammatory mediator, which can regulate the expression of several proteins involved in processes, such as glyceroneogenesis, adipocyte differentiation, de novo fatty acid synthesis and esterification [1]. TNF-α is known to inhibit the activity peroxisome proliferator-activated receptor gamma (PPARγ), the major transcriptional regulator of lipid and glucose metabolism [1,2]. TNF-α overexpression has been strictly correlated to adipose tissue dysfunction resulting in inflammation, augmented lipolysis, impairment of glucose uptake and insulin resistance (IR) [3]. IR has been described as a chronic low-grade inflammatory state in which insulin activity is impaired in adipose tissue. Since the discovery of its role in obesity-linked insulin resistance, TNF-α has been used to reproduce in vitro models of insulin resistance [4]. Lemon (*Citrus limon* (L.) *Burm. f.*) belongs to the Rutaceae family, which consists of dicotyledonous flowering plants that mainly grow in tropical and subtropical areas. According to genetics studies, lemon can be considered a hybrid between *Citrus aurantium* and *Citrus medica* [5]. Lemon is well known to have many beneficial effects on human health due to its rich content in bioflavonoids and other bioactive compounds, such as phenolic acids, organic acids, essential oils, vitamins, minerals, carotenoids and pectins [6]. Several studies [7,8] have assessed that polyphenols possess antioxidant and anti-inflammatory properties, in particular it has been shown the flavonoids ability to improve insulin-sensitivity and regulate lipid metabolism. Aim of this study was to evaluate lemon extract capability to restore adipocyte functions and insulin sensitivity in a model of TNF-α-induced insulin resistance.

## 2. Results and Discussion

The insulin resistance condition caused by TNF-α determined a decrease in PPAR-γ, insulin-regulated glucose transporter GLUT4 and fatty acid esterification simultaneously [9,10]. 

Several studies have shed light on the role of PPAR-γ in mediating bioactive compound activity and implications in improving peripheral tissues’ insulin sensitivity as well as lowering blood glucose levels [11,12]. Natural products have been used for centuries in the treatment of diabetes; in particular, many flavonoids contained in citrus fruits have shown potential antidiabetic effects due to their ability to reduce oxidative stress, improve glucose tolerance, modulate adipocyte differentiation and lipid metabolism and suppress inflammation [13,14,15,16].

**Figure 1:**
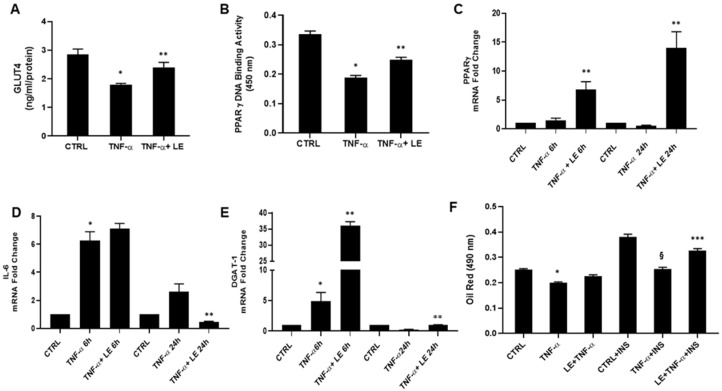
(**A**) Quantitative measurement of GLUT4 in cultured adipocytes treated with 100 ng/mL of TNF-α and 0.56 mg/mL of LE; (**B**) PPARγ transcription factor DNA binding activity in nuclear extracts; (**C**–**E**) RT-qPCR for PPARγ, IL-6 and DGAT-1 gene expression; (**F**) oil red O Staining in absence and presence of insulin (50 ng/mL). * *p* < 0.0001 CTRL vs. TNF-α, ** *p* < 0.005 TNF-α vs. TNF-α + LE, *** *p* < 0.0001 TNF-α + INS vs. LE + TNF-α + INS, ^§^ *p* < 0.0001 CTRL + INS vs. TNF-α + INS.

In our experimental conditions, protein quantification showed a 37% reduction in glucose transporter GLUT4 levels in TNF-α-treated cells compared to control; however the effect was reversed by LE pre-treatment (Figure 1A). In addition to its primary role as a regulator of adipocyte differentiation, PPAR-γ also activates genes involved in the lipogenic pathway and insulin signaling. Further investigations were carried out analyzing transcription factor PPAR-γ binding activity and gene expression level, using the PPAR-γ transcription factor assay and real-time quantitative PCR, respectively. As shown in Figure 1B, differentiated 3T3-L1 cells exposed to TNF-α (100 ng/mL) for 48 h displayed a decrease in PPAR-γ transcriptional activity, which was significantly improved by LE treatment, confirming lemon’s ability to restore insulin sensitivity and consequently adipocyte function. mRNA analysis at 6 and 24 h confirmed LE’s capability to increase PPAR-γ gene expression (Figure 1C). Recent studies report that IL-6 increases free fatty acid release from adipocytes, leading to hepatic steatosis and insulin resistance [17,18]. Following a 6 h treatment with the extract, IL-6 mRNA levels were similar to those of the TNF-α treated group, but a significant reduction following 24 h LE treatment was observed (Figure 1D). In order to obtain a complete overview of the insulin resistance condition, mRNA levels were also analyzed for the DGAT-1 (diacylglycerol o-acyltransferase 1) gene, a key metabolic enzyme responsible for the formation of triglycerides from diacylglycerol and Acyl-CoA [19]. After 6 h, DGAT-1 gene expression was considerably increased in the LE-treated group compared to that in the TNF-α-treated group, suggesting augmented synthesis of cholesterol, triglyceride accumulation and, thus, recovery of adipocyte function as well as balance between TNF-α-mediated lipolysis, lipogenesis and fatty acid esterification. The results after 24 h LE treatment showed a reinstatement of DGAT-1 levels comparable to those of the control group (Figure 1E). In order to verify the restored adipocyte function and lipid accumulation, oil red O staining was performed at the same experimental conditions as those of the abovementioned tests and also in the presence of insulin 50 ng/mL for a further 72 h to simulate IR conditions, characterized by elevated insulin levels and adipose tissue inflammation. Oil red O staining results showed a slight, but not statistically significant, increase in lipid accumulation in LE-treated cells compared to the TNF-α group. As expected, control cells exposed to insulin displayed an increase in lipid droplet formation, which was blocked by TNF-α treatment (Figure 1F). Interestingly, adipocyte LE exposure resulted in a significant increase in oil red quantification, demonstrating the high capability of this specific lemon extract to restore adipocyte insulin sensitivity. 

## 3. Materials and Methods

### 3.1. Characterization of Lemon Extract

VERDELLIMON^®^ dry powdered extract (batch no. 1/18), manufactured by Medinutrex (Catania, Italy) and standardized to contain ≥20.0% total flavanones and ≥1.0% total hydroxycinnamic acids (both by HPLC method), was used as LE for the assays as previously published. The effect of different concentrations on cell viability was previously tested, and LE 0.56 mg/mL was selected as the ideal and non-toxic concentration for the following experiments [20]. 

### 3.2. 3T3-L1 Cultured Cells, Adipocyte Differentiation and Induction of Insulin Resistance

The murine pre-adipocyte cell line 3T3-L1 was purchased from American Type Culture Collection (ATCC; Rockville, MD, USA) and grown in Dulbecco’s modified Eagle’s medium (DMEM) high glucose (HG) supplemented with 10% FBS and 1% penicillin–streptomycin and maintained at 37 °C and 5% CO_2_. Upon reaching confluence, we proceeded with a 5-day differentiation protocol using adipogenic medium [21] followed by 24 h of starvation. Insulin resistance was induced in a sub-cultured cell group using 100 ng/mL of TNF-α. Shortly after cells were starved, they were pre-treated with 0.56 mg/mL of LE, cultured for 48 h and then exposed to 100 ng/mL of TNF-α and 0.56 mg/mL of LE for 48 h.

### 3.3. Quantitative Measurement of Glucose Transporter 4 (GLUT4)

The Mouse Glucose Transporter 4 (GLUT4) Elisa kit (code n. MBS459629 Jiangsu, Suzhou, China) was used to quantitatively determine the transporter’s level in cell lysates. The plate provided in the kit was pre-coated with a specific GLUT4 antibody. Samples were set in the plate with a biotin-conjugated antibody solution specific to GLUT4, and then a reagent conjugated to horseradish peroxidase (HRP) was added to each well and incubated for an hour at 37 °C. After incubation wells were washed with a specific washing buffer provided by the manufacturer, the TMB substrate solution was added. Finally, an acid solution was used to stop the reaction resulting in a color change that was spectrophotometrically measured with a microplate reader at λ = 450 nm. The concentration of GLUT4 was expressed as ng/mL/total protein.

### 3.4. Quantitative Measurement of Peroxisome Proliferator-Activated Receptors Gamma (PPAR-γ)

PPARγ Transcription Factor Assay (code n. 10,008,878 Cayman, Ann Arbor, MI, USA) was used for the detection of specific transcription factor DNA binding activity in the nuclear extract. Briefly, the nuclear extract was obtained using NE-PER Nuclear and Cytoplasmic Extraction Reagents (code n. 78,833 Thermo Scientific, Rockford, IL, USA). The protocol requires the addition of two reagents to a cell pellet, causing cell membrane disruption and release of cytoplasmic content; then, the nuclei can be collected after centrifugation, and it is lysed with another reagent to obtain the nuclear extract. PPARs contained in the nuclear extract were detected using specific primary antibodies, and, subsequently, the addition of an HRP-conjugated secondary antibody resulted in a color development detectable at λ = 450 nm.

### 3.5. RNA Extraction and Quantitative Real-Time PCR Analysis

Briefly, upon reaching confluence, cells were harvested, and RNA extraction was per-formed using the Trizol reagent (Invitrogen, Carlsbad, CA, USA). First-strand cDNA was then synthesized with the Applied Biosystem (Foster City, CA, USA) reverse transcription reagent. Real-time quantitative PCR analysis was performed in 7900HT Fast Real-Time PCR System Applied Biosystems (Thermo Fisher Scientific, Waltham, MA, USA) using the SYBR Green PCR MasterMix (Life Technologies, Waltham, MA, USA) to evaluate different gene expressions linked to the insulin-resistance conditions. 

In particular, IL-6, PPARγ and DGAT-1 levels were evaluated after 6 and 24 h of treatment. The results were normalized with the housekeeping gene GAPDH using the comparative 2^−ΔΔCt^ method.

### 3.6. Oil Red O Staining

Oil Red O Staining was performed on insulin-resistant cells obtained using the abovementioned model with 100 ng/mL of TNF-α and in the presence of 50 ng/mL of insulin for a further 72 h. Cells were seeded in a 24 well white plate in 100 µL of DMEM high glucose (HG) supplemented with 10% FBS. OD was measured at λ = 490 nm, and lipid droplet accumulation was assessed using a microplate reader (Biotek, Synergy HT, Winooski, VT, USA).

### 3.7. Statistical Analysis

Statistical significance (*p* < 0.05) of differences between experimental groups was determined by the Fisher method for analysis of multiple comparisons. For comparisons between treatment groups, the null hypothesis was tested by either single-factor analysis of variance (ANOVA) for multiple groups or the unpaired t-test for two groups, and the data are presented as mean ± SEM.

## 4. Conclusions

To date, only few clinically approved drugs are used to treat diabetes, but an increasing number of natural product-derived compounds demonstrate antidiabetic effects. In this study, LE positively affects adipocyte metabolism by increasing nuclear receptor PPAR-γ levels, re-establishing adipocyte physiological functions and protecting 3T3-L1 cells against TNF-α-induced lipolysis. 

LE can be used for therapeutic approaches to prevent diabetes-associated inflammation, which leads to a pathologically insufficient capacity of adipose tissue to expand. Taken together, our results and scientific literature findings suggest that natural compounds may represent a valid alternative treatment to the multiple drugs used in conventional therapy.
